# Research on FBG Tactile Sensing Shape Recognition Based on Convolutional Neural Network

**DOI:** 10.3390/s24134087

**Published:** 2024-06-24

**Authors:** Guan Lu, Zhihui Shen, Ting Cai, Yiming Xu

**Affiliations:** 1 School of Mechanical Engineering, Nantong University, Nantong 226000, China; luguan@ntu.edu.cn (G.L.); george_sim@163.com (Z.S.); soulshinewas@ntu.edu.cn (T.C.); 2 School of Electrical Engineering and Automation, Nantong University, Nantong 226000, China

**Keywords:** fiber Bragg grating, tactile sensing array, flexible skin, convolutional neural network, shape recognition

## Abstract

Shape recognition plays a significant role in the field of robot perception. In view of the low efficiency and few types of shape recognition of the fiber tactile sensor applied to flexible skin, a convolutional-neural-network-based FBG tactile sensing array shape recognition method was proposed. Firstly, a sensing array was fabricated using flexible resin and 3D printing technology. Secondly, a shape recognition system based on the tactile sensing array was constructed to collect shape data. Finally, shape classification recognition was performed using convolutional neural network, random forest, support vector machine, and k-nearest neighbor. The results indicate that the tactile sensing array exhibits good sensitivity and perception capability. The shape recognition accuracy of convolutional neural network is 96.58%, which is 6.11%, 9.44%, and 12.01% higher than that of random forest, k-nearest neighbor, and support vector machine. Its F1 is 96.95%, which is 6.3%, 8.73%, and 11.94% higher than random forest, k-nearest neighbor, and support vector machine. The research of FBG shape sensing array based on convolutional neural network provides an experimental basis for shape perception of flexible tactile sensing.

## 1. Introduction

Realizing two-dimensional shape perception based on tactility has always been at the forefront of robot research. At present, shape recognition is mostly based on vision. With the maturity of imaging technology, researchers have conducted in-depth research on visual recognition and achieved good results [1,2]. In recent years, tactile recognition, as another direction of robot perception, has gradually attracted attention. As the direct contact perception method, tactile can obtain the material, size, weight, roughness, and other information of the measured object, which is an effective means of intelligent perception of robots.

Flexible skin made on the basis of a tactile sensor is an important carrier of robot perception. According to the manufacturing method and process, the tactile sensor can be divided into piezoelectric, varistor, capacitive, optical waveguide, etc. [3,4]. Tactile sensors are based on electromagnetism, with good precision and spatial resolution; however, they are not suitable for all scenarios, such as those with electromagnetic interference, high humidity, or corrosive environments. In response to this situation, optical sensors with anti-interference ability and corrosion resistance have been widely used. The optical tactile sensor has good reliability and a wide sensing range. As the kind of optical sensor, fiber Bragg grating sensors, in addition to the characteristics of general optical sensors, also have the advantages of small size, plastic geometry, and wide measurement range. In recent years, as a flexible tactile sensor, the FBG sensor has become a new hot spot in research [5,6]. At present, researchers at home and abroad have conducted in-depth research on FBG sensing technology and flexible skin. Jiang X. [7] used the change in the central wavelength and reflection spectrum, the total power of the reflection spectrum, and the peak power to distinguish the plane, edges, and angles. The FBG flexible skin made by Qian M. et al. [8] uses heat transfer to induce wavelength changes to distinguish between aluminum, iron, and plastic. Wang J. [9] made an FBG sensing array and used a neural network (ANN) to recognize circles, squares, and triangles with an accuracy of 88%. Wang Y. et al. [10] used the random forest algorithm to identify the perceptual slip signal of FBG flexible skin; they realized the recognition of two features of slip sense and found certain value in the research of sliding signals in the field of flexible bionic skin sensing. Schneebeli et al. [11] embedded FBG in rubber carpets and used the k-nearest neighbor (KNN) algorithm to realize the recognition and classification of two-dimensional shapes. This can provide a basis for the study of human gait. At present, a few studies have been conducted on shape recognition using the FBG tactile sensing array applied to flexible skin. In some studies, there are few types of shape recognition, and there are defects in recognition accuracy and efficiency. On the other hand, the selected algorithm objectively has the disadvantages of slow convergence, easily falling into local optimization, and the need to debug a large number of parameters, thus affecting the success rate of final identification.

Traditional machine learning algorithms rely on expert experience for feature selection and extraction. The generalization ability is limited, and it is easy to produce dimensional disasters. Therefore, deep learning has gradually become a hot research issue. As an excellent model in deep learning, the convolutional neural network (CNN) is widely used in classification recognition, overcomes the shortcomings of traditional classification recognition methods, can automatically learn valuable features from the original data, and largely gets rid of expert experience. CNN can process a large amount of data, and effectively extract data features and classify them [12].

As a sensitive element, fiber gratings are easily damaged and require encapsulation protection [13].Currently, the commonly used method for producing packaging layers is the molding method. The steps of this method are as follows: First, a metal or plastic mold is designed according to the shape of the sensor; Secondly, the encapsulation layer solution is injected into the mold; Then, put the mold into the baking box to bake it into shape; Finally, the mold is removed to obtain the packing layer of sensor. Because this method is performed manually, it is easily affected by factors such as manufacturing accuracy and molding efficiency. The application of 3D printing technology has changed the previous packaging of bare fiber gratings, which can improve the preparation efficiency and accuracy [14]. Currently, the packaging materials used for flexible skin mainly include silicone and polydimethylsiloxane (PDMS) [15]. PDMS has good flexibility and plasticity (Young’s modulus is 9.2 Mpa) and has been widely used as a flexible skin material [16,17]. Due to the long curing time of PDMS, flexible resin can be used to replace PDMS when 3D printing the encapsulation layers of flexible sensors. Flexible resin (Flexible 80A, Formlabs, Somerville, MA, USA) [18], as a new type of industrial resin, has flexibility, elasticity, and excellent crack resistance [19]. After research, the parameters of this material are close to PDMS, and it is suitable as a flexible encapsulation material of FBG. More importantly, it can be directly used for 3D printing.

Aiming at the shortage of production and recognition efficiency of shape perception sensors applied to flexible skin, this paper proposes an FBG tactile sensing array shape recognition method based on CNN, which uses 3D printing technology to make FBG tactile sensing array; collects wavelength data of different shapes; and compares the results of CNN, random forest (RF), support vector machine (SVM), and k-nearest neighbor (KNN), verifying the feasibility and reliability of the sensing array tactile perception and shape recognition method.

## 2. The Principle of FBG Sensing and Shape Recognition Algorithm

### 2.1. Sensing Characteristics of FBG

**Figure 1:**
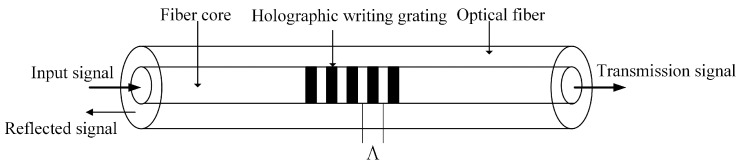
Schematic diagram of FBG.

Fiber Bragg grating is a photonic device with a periodic distribution of physical structure in the optical fiber guide medium and used for wavelength selection by the starting band-blocking filter [20]. The structural diagram and reflection transmission characteristics of FBG are shown in Figure 1.

According to the coupling mode theory, when broadband light is transmitted in FBG, mode coupling will be generated, and the light that meets the fiber Bragg condition will be reflected. The central wavelength of FBG is

(1) $$\lambda_{B}=2n_{eff}\Lambda$$

In the formula, $\lambda_{B}$ is the central wavelength of FBG, $n_{eff}$ is the effective refractive index of the reverse coupling mode, and Λ is the grating period. From Formula (1), it can be seen that the central wavelength of FBG, is determined by the variables $n_{eff}$ and Λ. When external factors cause these two parameters to change, the central wavelength of FBG will change accordingly. Among all external factors, temperature and strain are the main factors causing changes in the wavelength of the FBG. The relationship between the central wavelength $\lambda_{B}$ of the fiber grating and the strain and temperature is as follows:

(2) $$\Delta \lambda_{B}=\lambda_{B}\left(1-\frac{n_{eff}^{2}[P_{12}-v\left(P_{11}+P_{12}\right)]}{2}\right)\varepsilon +\left(\alpha +\xi \right)\Delta T$$

In the formula, ε is the strain of FBG, α is the thermal expansion coefficient of the fiber, ξ is the thermo-optical coefficient, ΔT is the temperature change, *v* is the Poisson ratio of the fiber core material, and $P_{1j}$ (*j* = 1, 2) is the Pockel coefficient. When the temperature does not change, only the strain change ε occurs in the above equation. Take $n_{eff}$ = 1.456, Poisson ratio *v* = 0.17, elastic coefficients $P_{11}$ = 0.121, $P_{12}$ = 0.270, and calculate the strain of FBG from Formula (2) to [20]:

(3) $$\Delta \lambda_{B}=0.784\lambda_{B}\varepsilon$$

### 2.2. Strain Transfer Rate of FBG

**Figure 2:**
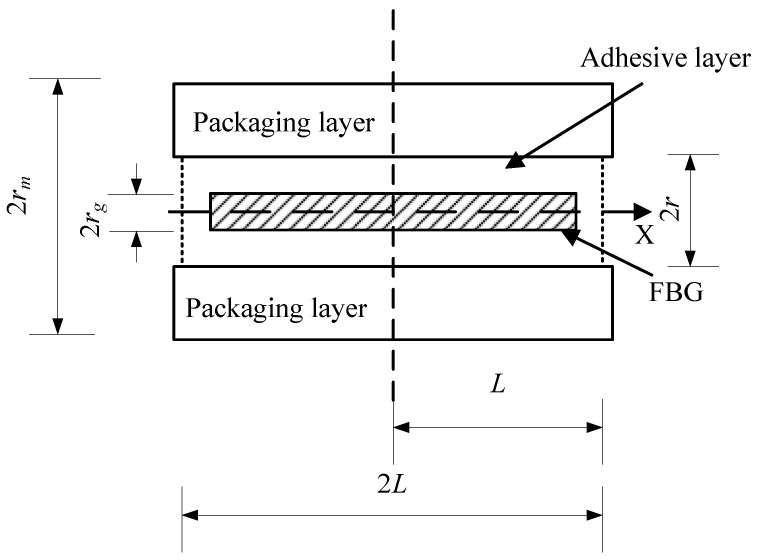
Sensor structure diagram under microscopic morphology.

The sensor model used in this paper is an optical fiber embedded structure, and the FBG sensor is approximately a symmetric structure. Its microstructure is shown in Figure 2.

In Figure 2, the shadow area is the grating part and $r_{g}$ is the radius of the fiber; the dotted line area is the adhesive layer, 2*L* is the total length of the cementing layer, *r* is the thickness of adhesive layer, and $r_{m}$ is the packing thickness. The formula for calculating the strain transfer rate of the axial points of the fiber is

(4) $$a=1-\frac{\cosh(\mathrm{kx})}{\cosh(\mathrm{kL})}$$

The average strain transfer rate of each axial point of the fiber is

(5) $$\bar{a}=1-\frac{\sinh(\mathrm{kL})}{kL\cosh(\mathrm{kL})}$$

In the formula: *a* is the strain transfer rate; *L* is the semi-adhesive length of FBG; $\bar{a}$ is the average strain transfer rate; and *k* is the parameter related to the characteristics of fiber and intermediate materials, given by the following formula:

(6) $$k^{2}=\frac{2G_{i}}{r_{g}^{2}E_{f}\ln(\frac{r}{r_{g}})}$$

In the formula, $E_{f}$ is the Young’s modulus of the fiber and $G_{i}$ is the shear modulus of the adhesive layer [21,22].

### 2.3. Introduction to the Principle of Algorithm

**Figure 3:**
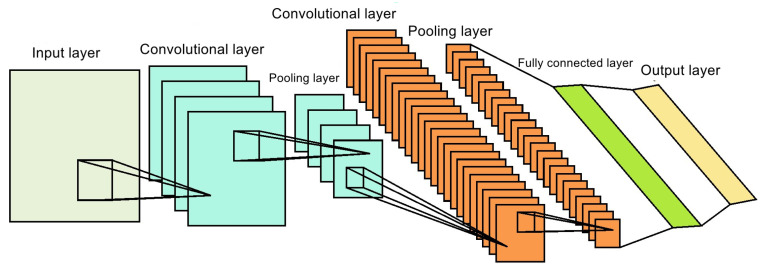
The structure of CNN.

CNN is different from traditional fully connected neural networks. CNN adopts convolutional layers and pooled layers, which reduce the parameters of the model and effectively reduce the risk of over-fitting of neural networks and the difficulty of training. CNN is mainly composed of input layers, convolutional layers, pooled layers, and fully connected layers [23], as shown in Figure 3.

The input layer of CNN can standardize multi-dimensional data, which helps improve the algorithm’s operational efficiency and learning performance. In the convolutional layer, the convolutional kernel performs convolutions on the output from the previous layer and uses a nonlinear activation function to construct the output features. The output of each layer is the result of convolving multiple input features, and its mathematical model is:

(7) $$y_{i}^{l+1}\left(j\right)=\omega_{i}^{l}\cdot x^{l}\left(j\right)+b_{i}^{l}$$

Among them, $\omega_{i}^{l}$ is the weight of the *i*-th filter nucleus in layer *l*; $b_{i}^{l}$ is the bias of the *i*-th filter core in layer 1; $x^{l}\left(j\right)$ is the input of the *j*-th neuron of the first layer; $y_{i}^{l+1}\left(j\right)$ is the input of the *j*-th neuron in layer *l* + 1; and the symbol “·” indicates the dot product of the kernel and the local area. After the convolution operation, the activation function performs a nonlinear transformation of the output of the logical value of each convolution. The function of the activation function is to transform the multidimensional features that are inseparable from the original line to another space and enhance the linear separability of these features. This paper uses the Relu function as the activation function. When the input value is greater than 0, the derivative of the function is always 1; so, the problem of gradient disappearance is overcome [24,25].

### 2.4. The Recognition Model Based on CNN

**Figure 4:**
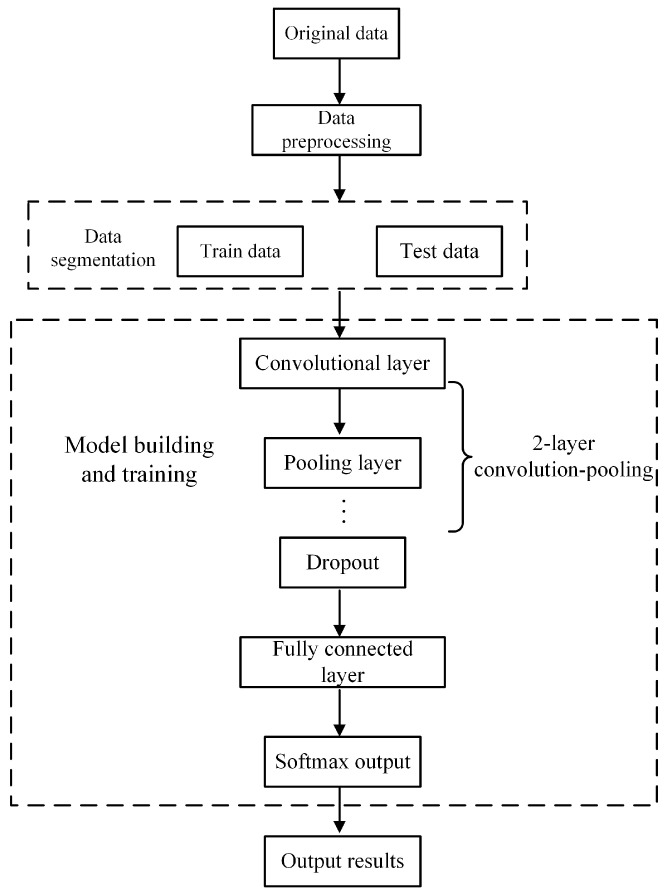
The process of CNN.

Based on the basic principles of the convolutional neural network, this paper built a one-dimensional convolutional neural network process, as shown in Figure 4. The specific steps were as follows:

1. Data collection, using FBG sensor to collect data of each shape;
2. Data preprocessing;
3. Divide the data into training sets and test sets, build a 1D-CNN model, and substitute the sample set data into the model for training;
4. Evaluate the model.

The processed data are imported into 1D-CNN. The 1D-CNN has a strong feature extraction ability, in which the features hidden in the original data can be automatically extracted alternately through the convolutional layer and the pooling layer, and the adaptive feature learning can be completed in the fully connected layer. In this way, the 1D-CNN algorithm eliminates the process of manually extracting features in the traditional algorithm and realizes end-to-end information processing. The specific structure of 1D-CNN proposed in this paper consists of two convolutional layers, two pooling layers, three fully connected layers, and one softmax output layer. After passing through the first layer convolutional layer, the signal is converted into a set of feature mappings, and then sampled by maximum pooling. After these operations are repeated, the characteristics of the last pooling layer are connected to the full connection layer; then, the full connection layer is activated through the Relu function and passed to the softmax layer; finally, the probability value of each classification is obtained, in which the category with the highest probability is regarded as the recognition result.

## 3. Simulation Analysis and Structural Design of FBG Sensor

The finite element simulation analysis was carried out in ANSYS, and the size was set as 100 mm × 50 mm × 5 mm, the elastic modulus was 8.9 Mpa, Poisson’s ratio was 0.41, and the density was 1.4 g/cm^3^. Set the path in X, Y, and Z directions to check the strain. During the simulation, vertical force is applied to the upper surface of the elastomer for static analysis, and the strain of each specific position can be viewed in the postprocessing module after solving it. The stress-strain curve of the material under unidirectional loading is obtained in the experiment, while in practice, the stress state of the structure is often a spatial stress state, and yielding also results in a spatial yield. The equivalent stress corresponding to the strain state after yielding and the spatial stress state of the structure at this time can be found according to the equivalent strain, in order to determine the buried depth of the fiber and the transverse and longitudinal dimensions between the adjacent fiber gratings.

### Analysis of Depth, Grating Spacing, and Optical Fiber Spacing of FBG

**Figure 5:**
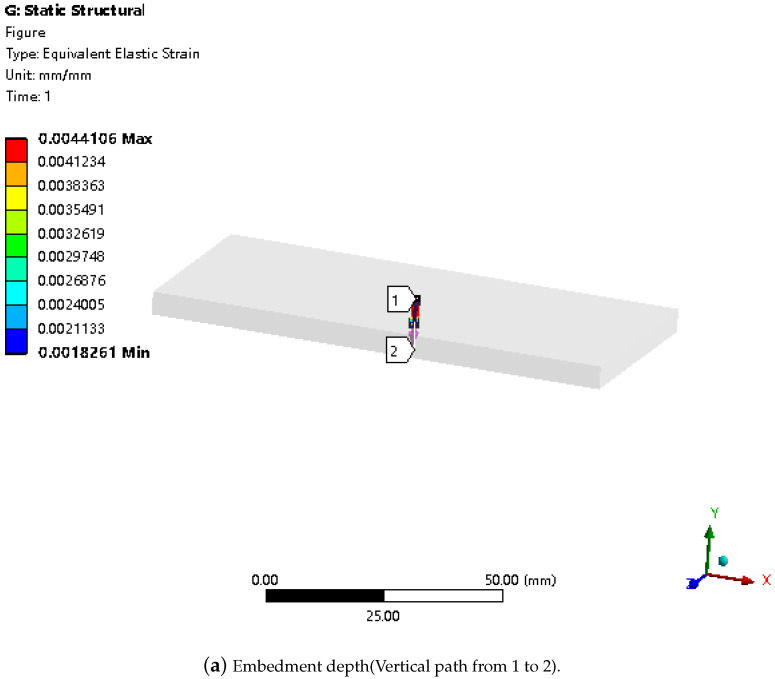

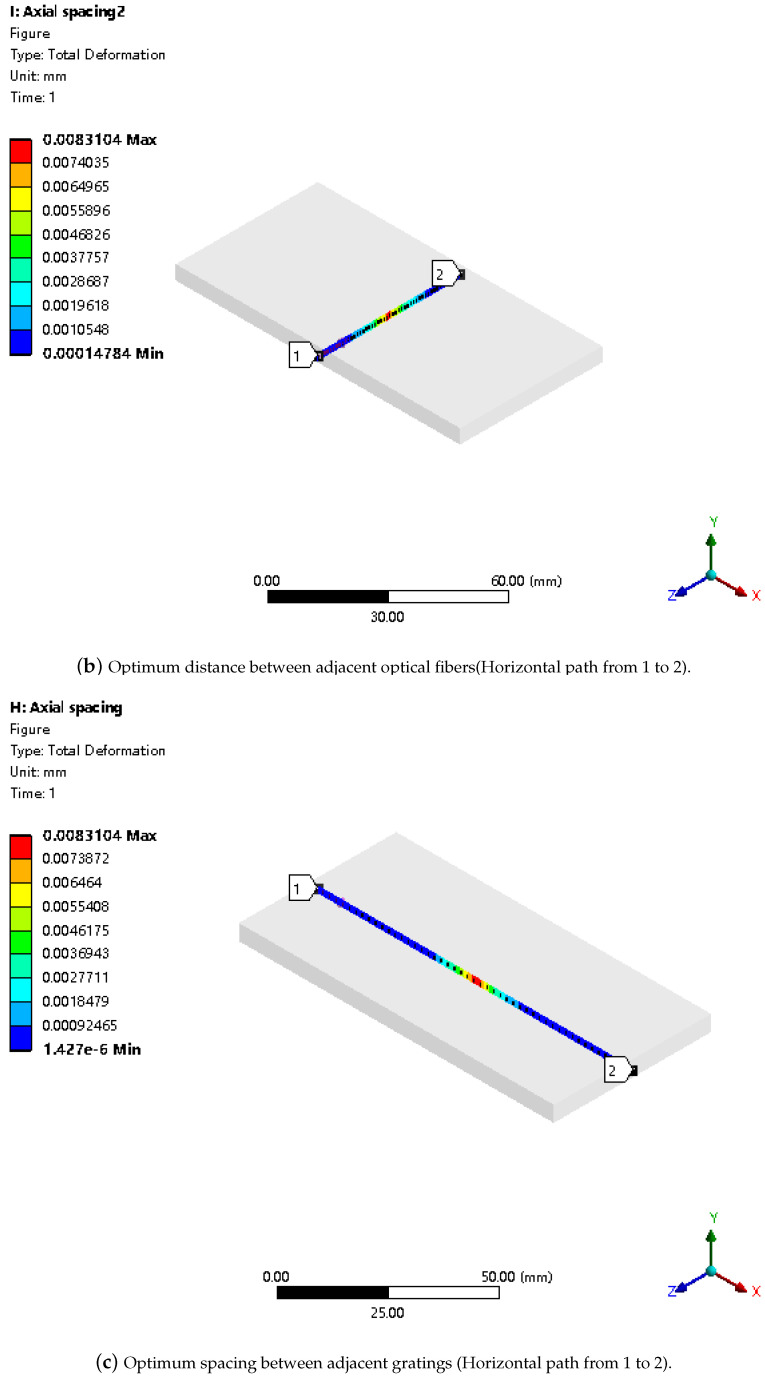
Ansys simulation analysis.

In ANSYS, the center of the lower surface of the elastomer was taken as the coordinate system and paths in three directions of X, Y, and Z were constructed. A 1 N vertical downward force was applied to the center of the upper surface of the elastomer. The most obvious area of strain on the three paths was the embedding depth of FBG, grating interval, and fiber interval data. The simulation and maximum strain range results are shown in Figure 5 and Table 1.

**Figure 6:**
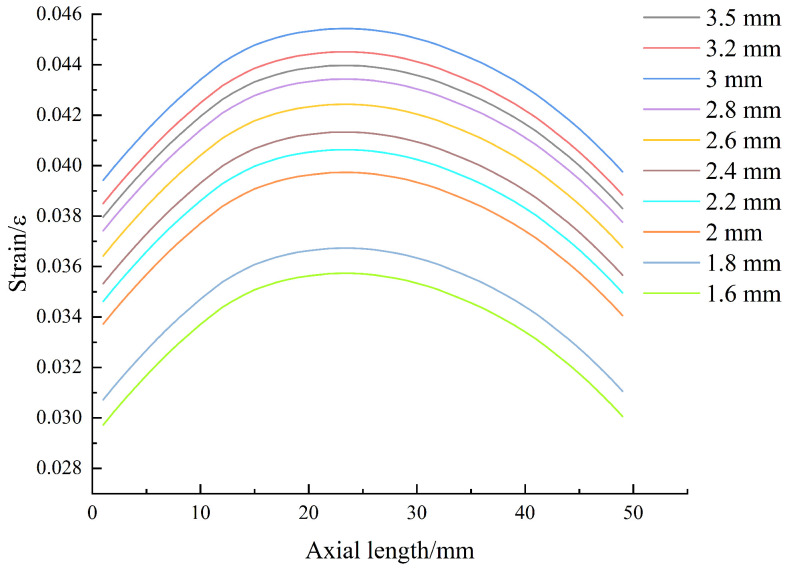
Axial strain at different embedment depths.

Since the embedding depth obtained by simulation ranges from 1.498 mm to 3.502 mm, in order to determine the final embedding depth, the X-axis axial path was set at the distances of 1.6 mm, 1.8 mm, 2 mm, 2.2 mm, 2.4 mm, 2.6 mm, 2.8 mm, 3 mm, 3.2 mm, and 3.5 mm from the upper surface of the model, and the path deformation data were checked. The path with the largest overall deformation is the best depth. Through simulation, the optimal embedding depth obtained is 3 mm, as shown in Figure 6.

**Figure 7:**
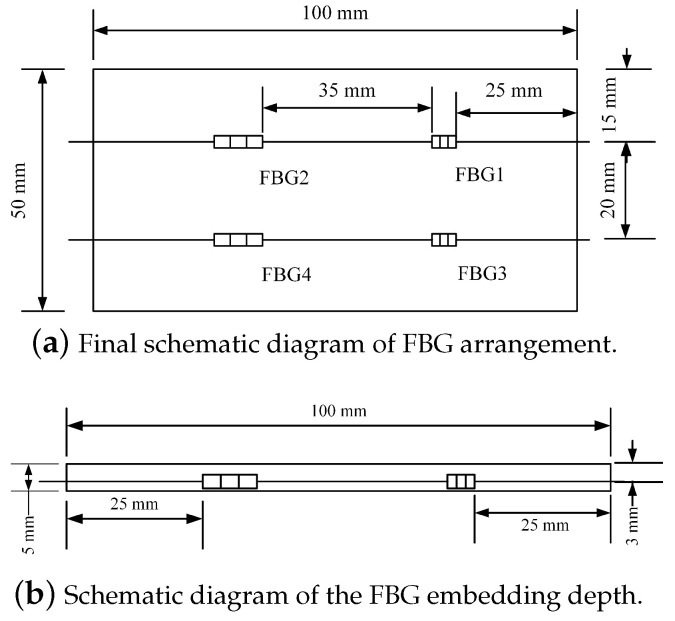
The schematic diagram of the FBG sensing array.

The schematic diagram of the designed FBG tactile shape sensing array is shown in Figure 7.

FBG is sensitive to strain and temperature at the same time. When the stress and strain parameters are monitored by FBG, the interference of temperature is inevitable. It is difficult to distinguish the stress or temperature caused by the last wavelength change. Therefore, compensation or differentiation must be made in the application. According to reference [26], the FBG sensing array in this paper is compensated for temperature by adding a reference grating.

**Figure 8:**
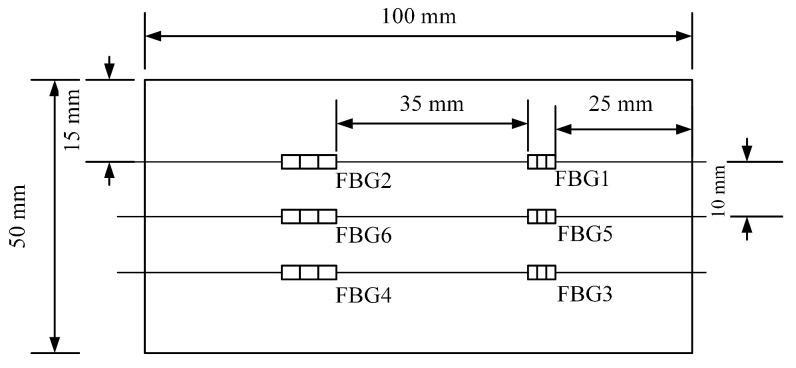
Final schematic diagram of the FBG sensing array.

The schematic diagram of the designed FBG sensing array is shown in Figure 8. FBG5 and FBG6 are used as reference gratings for temperature compensation.

## 4. Sensitivity Experiment of FBG Tactile Sensing Array

### 4.1. Production of FBG Tactile Sensing Array

According to the structure of the FBG sensing array designed above, in order to better compare the experimental results and analyze the experimental characteristics of fiber with different grating lengths, temperature compensation, and demodulation, different fibers were selected when making the FBG tactile shape sensing array. The parameters of FBGs selected for the final experiment are shown in Table 2, which were purchased from Technica Co., Ltd. (Beijing, China).

In the process of burying the optical fiber into the packaging layer, it needs to be fixed with glue. However, due to the differences in the physical properties of the optical fiber, glue, and packaging layer, the strains of each material are not equal; so, the strain measured through FBG is not the actual strain of the sensor. Therefore, the grating bonding layer needs to be analyzed to ensure that the FBG sensor has the best sensing performance.

In the three-layer strain transfer model of the fiber core–colloid–encapsulation layer, the reserved hole is filled with adhesive and the size is equivalent to the thickness of the glue layer. In order to facilitate the embedding of FBG, the package layer was divided into 2.8 mm thickness and 2.2 mm thickness during 3D printing. Because the FBG embedding depth is 3 mm, the design reserved hole radius *r* = 0.2 mm.

**Figure 9:**
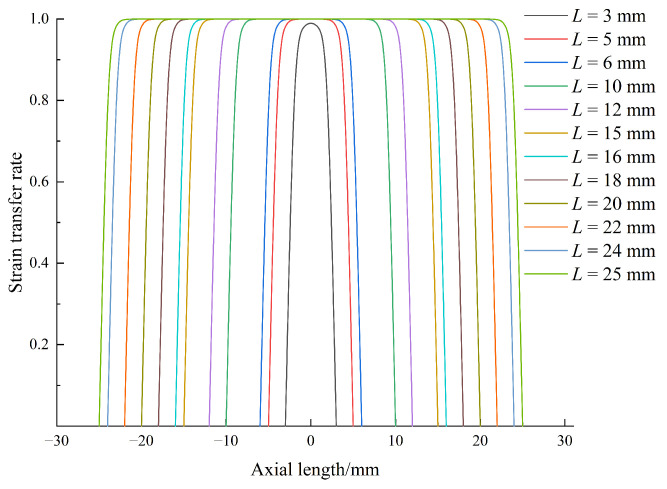
Relationship between bonding length and strain.

In this paper, the fiber’s radius $r_{g}$ = 0.0625 mm, the fiber’s Young’s modulus $E_{f}$ = 72,000 MPa, and the reserved hole *r* = 0.2 mm. According to the literature [27], the $G_{i}$ of the selected glue = 1350 MPa. When *L* = 3 mm, 5 mm, 6 mm, 8 mm, 10 mm, 12 mm, 15 mm, 18 mm, 20 mm, 22 mm, 24 mm, and 25 mm, draw the image, as shown in Figure 9.

All strain transfer coefficient curves are axisymmetrically distributed with respect to x = 0, and all strain transfer coefficient curves plunge to 0 at x = ±*L*. At the same time, the maximum value of the curve with *L* = 3 mm is only obtained at x = 0 and is less than 1. For the remaining adhesive total length, the optimal value 1 is obtained within the range around x = 0. The reason is that *L* = 3 mm is too small, resulting in a lower strain transfer coefficient than the other conditions. It can also be observed from the curve distribution that the downward trend of all curves near x = ±*L* is almost the same because the elastic modulus of the rubber layer is the same. The difference is that the range of the optimal strain transfer coefficient can be maintained and increases with the increase in the adhesive length of 2*L*. Therefore, it can be concluded that the effective adhesive length should be extended as far as possible when attaching the fixed fiber so that the optimal strain transfer coefficient can completely cover the sensing length of the fiber along the distribution range of the adhesive layer length. In this paper, the sensing unit on the fiber is the grating segment, and its length is no more than 10 mm, so the effective bonding length of at least 20 mm (*L* = 10 mm) must be ensured to achieve the ideal strain transfer effect.

**Figure 10:**
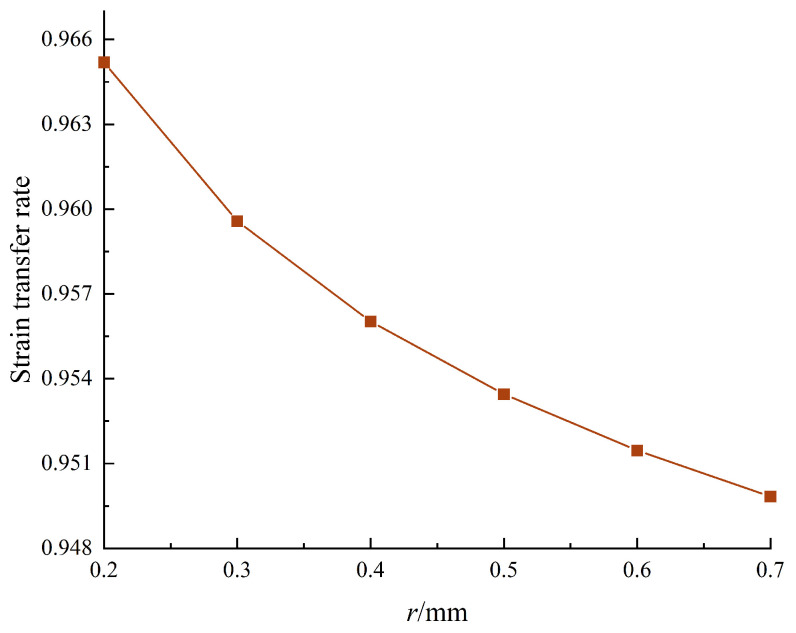
Reserved hole radius and strain rate.

The influence of reserved hole radius on the strain transmissibility of FBG is shown in Figure 10. It can be seen from the figure that the strain transfer rate decreases with the gradual increase in the size of the reserved hole. Therefore, setting the reserved hole *r* = 0.2 mm can meet the requirements.

**Figure 11:**
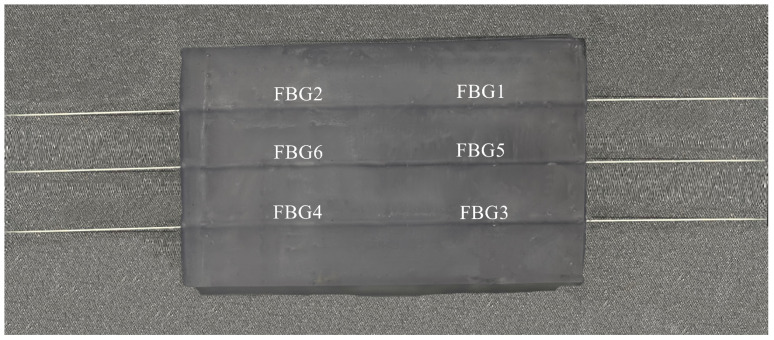
FBG tactile shape sensing array.

Flexible resin was chosen for 3D printing. FBG was buried in the through hole and fixed with glue. The paste length of a single optical fiber was 20 mm (*L* = 10 mm), and the two packaging layers were also fixed with glue. Finally, the physical sensing array is shown in Figure 11.

### 4.2. Sensitivity Calibration Experiment of FBG Tactile Sensing Array

**Figure 12:**
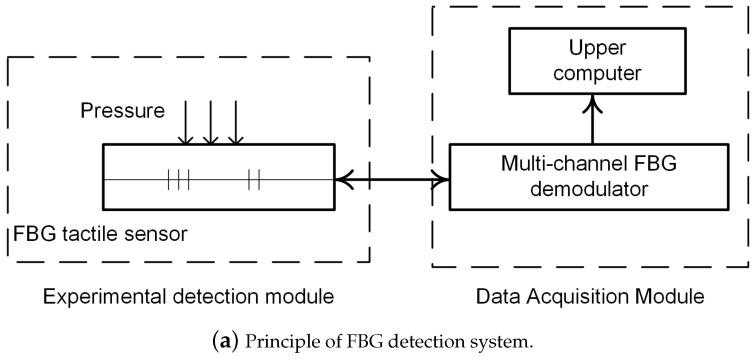

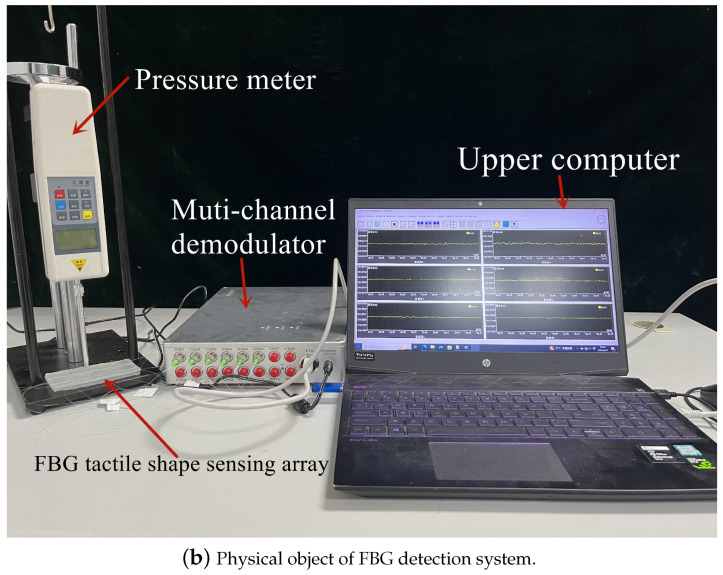
Principle and physical diagram of FBG detection system.

A calibration experimental system was built to detect the sensitivity of the designed FBG tactile shape sensing array. The experimental principle and built experimental system are shown in Figure 12. The whole system included FBG tactile shape sensing array, pressure meter, multi-channel demodulator, and upper computer. By collecting the output signals of multiple discrete points on the surface of the FBG sensing array packaging material, the contact information between the tactile sensor and the object was detected. The demodulation instrument has 16 channels, the demodulation wavelength range is 1525–1567 nm, and the resolution is 0.1 pm. Because the sensing array is bending, it is difficult for the press to apply the corresponding load, and under bending conditions, it will cause certain interference to the central wavelength of FBG. In order to ensure that the FBG tactile shape sensing array can effectively sense the external load, the experiment was carried out on a flat desktop.

**Figure 13:**
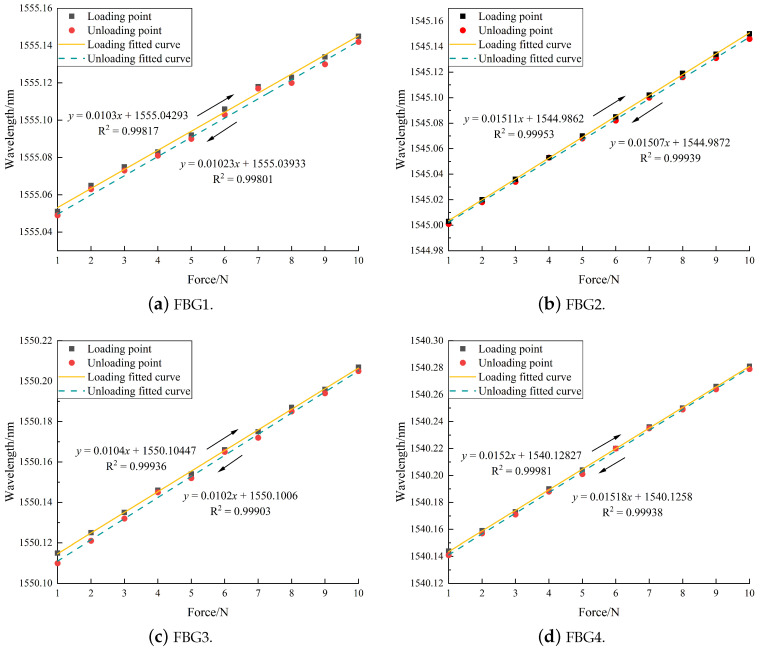

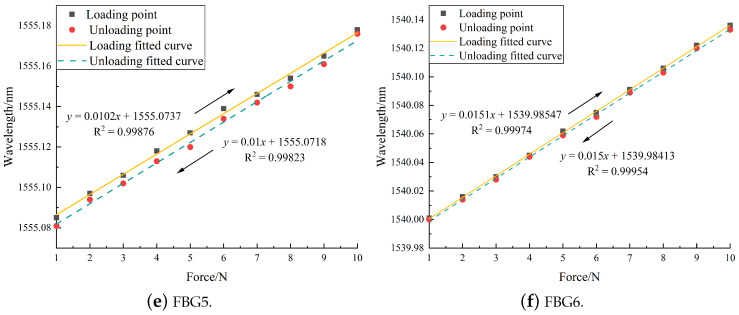
Sensitivity of FBG1-FBG6.

Contact force was applied directly above each FBG. The range of force was 1–10 N, and the applied step was 1 N. The experiment was carried out 5 times, and the average wavelength of these 5 experiments was obtained. Through experiments, the sensitivity of loading and unloading was obtained, as shown in Figure 13. The temperature was kept constant during the experiment.

It can be drawn from Figure 13 that the response curve of FBG has obvious changes. From the sensitivity and fitting excellence data, it can be concluded that FBG can accurately sense the contact force applied above the position. The average sensitivity of FBG with a grid length of 10 mm is 15.105 pm/N, and the average sensitivity of FBG with a grid length of 5 mm is 10.24 pm/N. The relative error of the sensitivity of loading and unloading a single FBG does not exceed 2%. At the same time, it can also be seen that the loading (unloading) sensitivity of the FBG with a long grating is higher than that of the FBG with a short grating.

### 4.3. Linearity

The ideal measurement system should have a linear output–input relationship with the same sensitivity throughout the measurement range. The ratio of the maximum difference between the experimental calibration curve of the sensor and the data fitting line and the full-scale output is the linearity of the sensor; so, the linearity is also called “nonlinear error”. The smaller the value, the better the linearity.

(8) $$e=\frac{\Delta H_{max}}{y}$$

In the formula, $\Delta H_{max}$ represents the maximum difference between the arithmetic mean value of the central wavelength of FBG in five cycle tests and the reference point value on each fitted line, and *y* is the wavelength offset of full-scale FBG. Table 3 shows the experimental linearity of 6 FBGs.

It can be seen from the table that the linearity of the 6 FBGs are all within 0.04, indicating that the central wavelength of the FBG tactile shape sensing array has a good linear relationship with the load size, and the overall change is relatively uniform, indicating that it has good tactile perception ability and the flexible resin material as an encapsulation material can ensure that the embedded fiber grating can effectively sense the external load.

## 5. Shape Recognition Based on FBG and CNN

### 5.1. Data Collection and Algorithm Parameters

**Figure 14:**
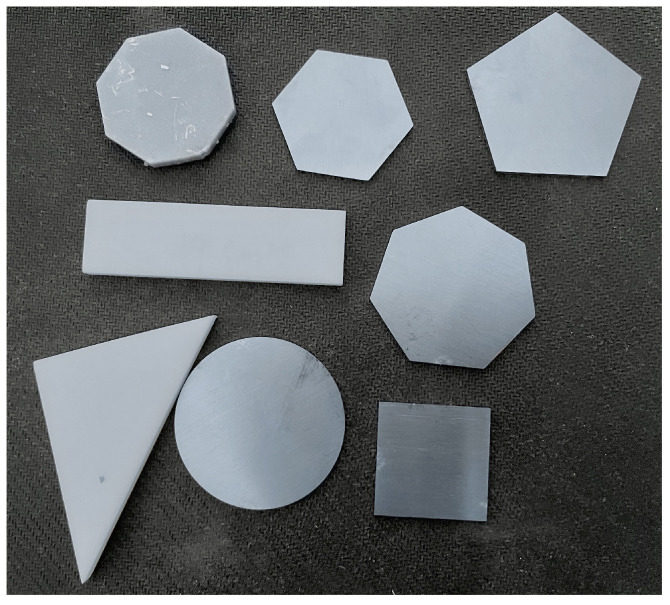
The specific shape selected for the experiment.

According to the application scenarios of FBG shape sensing array such as production line assembly, rehabilitation medical treatment, and virtual reality, the commonly used shape types such as triangle, circle, rectangle, square, and polygon are selected. The specific information of objects is shown in Table 4 and Figure 14, and their height is 3 mm. These shapes were placed on the sensing array, and the force of 1–15 N was applied using the pressure meter to collect the central wavelength. The temperature is kept constant during the experiment.

The collected data need to be normalized. The purpose of normalization is to adjust the data to a certain numerical range, usually to eliminate dimensional differences, avoid weight imbalance, and improve the convergence speed of the model. The equation for normalization is as follows:

(9) $$x_{new}=\frac{x-x_{min}}{x_{max}-x_{min}}$$

Among them, *x* represents the original data, $x_{min}$ is the minimum value of the data, $x_{max}$ is the maximum value of the data, and $x_{new}$ is the normalized value.

Data were collected through the FBG tactile sensing array system. The raw data were processed so that the samples were a 3000 × 1 vector, and the vector was input into the CNN. After two fully connected layers, eight neurons were outputted, which represented eight shapes, respectively. According to the output of eight neurons, the shape type to which the sample belongs can be confirmed. The learning rate was set to 0.001 and the epoch was set to 500. The parameters established are specified in Table 5.

The evaluation indicators of the model generally include accuracy, precision, recall, and F1 score. Accuracy denotes predicting the correct results as a percentage of the total samples, which can directly reflect the effect of recognition. The precision represents the proportion of true positive cases in the prediction result; the recall represents the percentage of all positive cases that are correctly predicted. In many cases, it is necessary to combine the two well. The F1 score can be seen as a weighted average of precision and recall, with higher values indicating better recognition results. The formula is as follows [28]:

(10) $$F1=\frac{2\times precision\times recall}{precision+recall}$$

### 5.2. Results and Analysis

**Figure 15:**
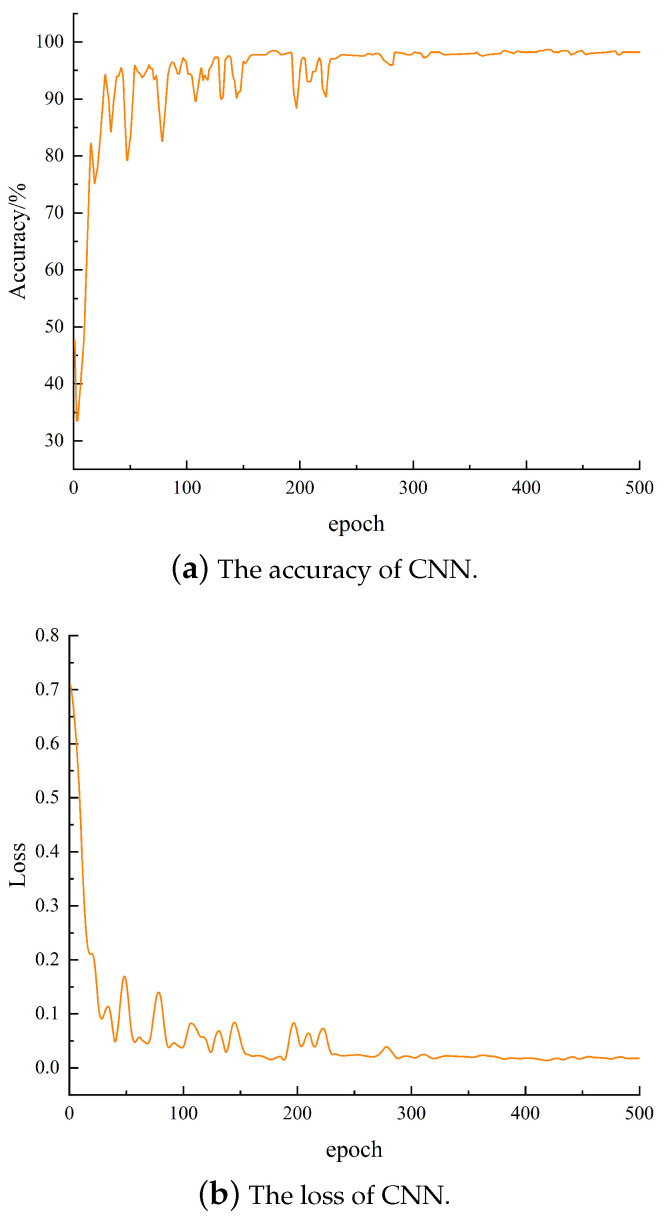
The accuracy and loss of CNN.

The data were divided into the training set and the test set at a 7:3 ratio and input into CNN for classification. The accuracy and loss values of the test set are shown in Figure 15. With the increase in iterations, the curve of accuracy rate and loss value gradually becomes stable, and the average recognition accuracy rate of CNN is 96.58%.

**Figure 16:**
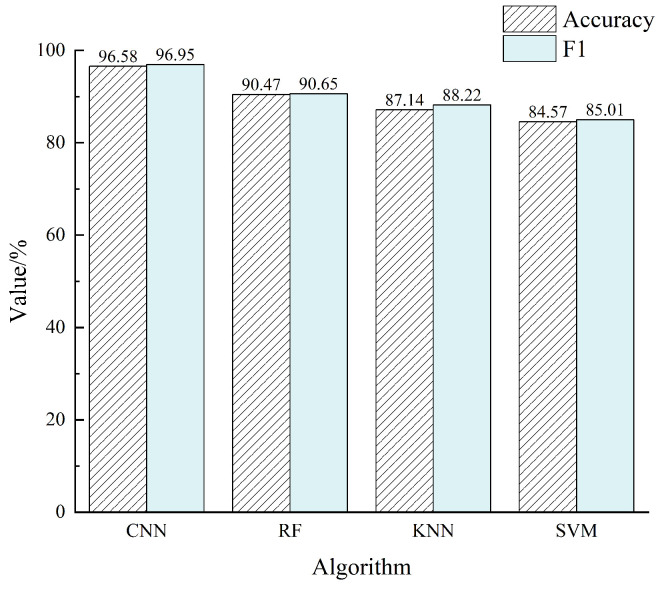
Accuracy and F1 score of the four algorithms.

In order to verify the performance of CNN recognition, the same experimental data were inputted into the three algorithms of RF, KNN, and SVM for classification identification. The average recognition accuracy and F1 score are shown in Figure 16.

CNN’s accuracy is 6.11%, 9.44%, and 12.01% higher than RF, KNN, and SVM, and its F1 score is 6.3%, 8.73%, and 11.94% higher than RF, KNN, and SVM. In this experiment, CNN’s recognition accuracy and F1 score are significantly higher than the other three algorithms.

### 5.3. The Number of Shapes and the Influence of Applied Contact Force on Recognition

In the above experiment, different contact forces were applied to eight shapes and recognition experiments were carried out. The classification accuracy and F1 score were obtained. This experiment will study the effects of the number of shapes and the applied contact force on CNN recognition accuracy and F1 score.

#### 5.3.1. The Influence of the Number of Shapes on Recognition

**Figure 17:**
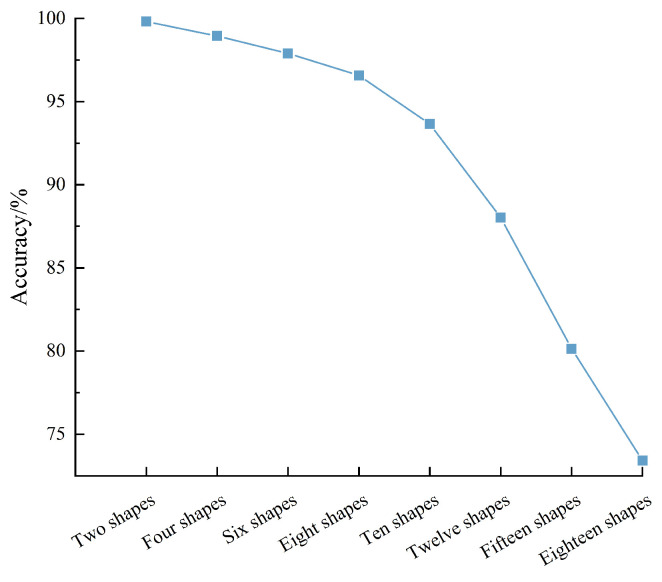
The influence of the number of shapes on the accuracy of CNN.

During the experiment, two shapes (square and rectangle), four shapes (square, circle, triangle, and pentagon), six shapes (square, rectangle, circle, triangle, pentagon, and heptagon), twelve shapes (square, rectangle, circle, triangle, pentagon, heptagon, octagon, hexagon, nonagon, parallelogram, trapezoid, and diamond), fifteen shapes (square, rectangle, circle, triangle, pentagon, heptagon, octagon, hexagon, nonagon, parallelogram, trapezoid, diamond, decagon, dodecagon, and semicircle), and eighteen shapes (square, rectangle, circle, triangle, pentagon, heptagon, octagon, hexagon, nonagon, parallelogram, trapezoid, diamond, decagon, dodecagon, semicircle, fan, circle, and oval) were selected for experimental comparison. Most FBG shape sensing array application scenarios cover these shape classes, which were selected for experimental comparison, and the contact force applied during the experiment was consistent. CNN was used to classify and identify the experiment, and the accuracy rate obtained is shown in Figure 17.

From Figure 17, the following can be seen:

1. As the number of shapes increases, the accuracy of CNN gradually decreases, indicating that the number of shapes has a certain impact on the recognition accuracy. The higher the number, the lower the accuracy.
2. The accuracy decline trend is divided into two stages. When the number of shapes is greater than 10, the accuracy curve declines faster and the recognition accuracy of 15 shapes is above 80%, which can meet the requirements of engineering applications.
3. At the same time, when there are 18 types of shapes, the accuracy is lower than 75%. The reason is that the more shapes, the higher the similarity, which has an impact on the final accuracy rate. In general, CNN has a high recognition rate for different shape types, and can distinguish trilateral, quadrilateral, circle, and polygon, which meets the application scenarios of FBG sensing array.

#### 5.3.2. The Influence of Contact Force on Recognition

**Figure 18:**
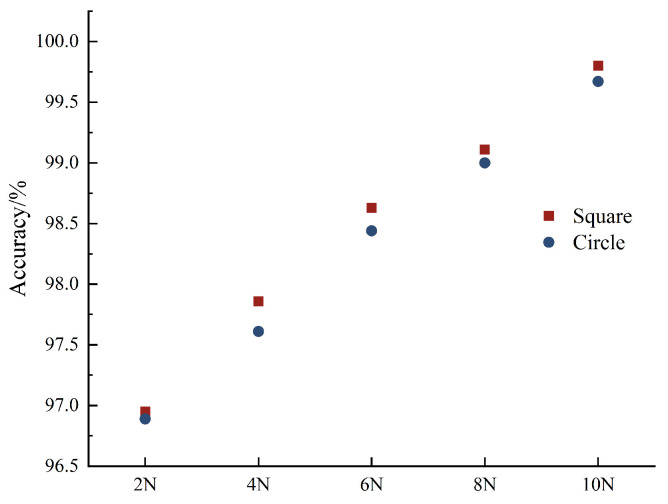
Accuracy of square and circle under different contact force values.

The influence of contact force on accuracy is studied by applying different contact force to the shape. During the experiment, a square and circle were selected for the experiment. The area data were consistent with Section 5.1, and the contact forces applied were 2 N, 4 N, 6 N, 8 N, and 10 N in turn. Under different contact forces, the accuracy of square recognition is 98.47%, and the accuracy of circle recognition is 98.31%. The accuracy of each applied load is shown in Figure 18.

As can be seen from the figure, with the increase in contact force, the accuracy of the recognition of the same shape also increases, indicating that the contact force will affect the recognition result. The greater the contact force, the better the recognition effect.

### 5.4. The Influence of Random Error on the Accuracy of the Algorithm

**Figure 19:**
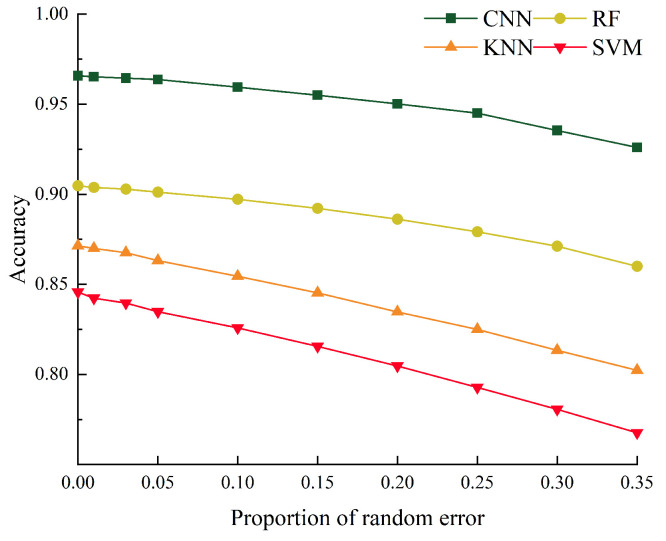
The influence of random error on the accuracy of four algorithms.

In practice, the FBG tactile shape sensing array may be affected by different noises when collecting corresponding data. In order to be more in line with engineering application scenarios, the wavelength data of eight shapes (square, rectangle, circle, triangle, pentagon, heptagon, octagon, and hexagon) were normalized, and random errors were added to them to test the anti-noise capability of the algorithm. The expression of random errors is as follows:

(11) $$X_{0}=\hat{X_{0}}+error\times \hat{X_{0}}\times unifrnd\left(-1,1,1,size\left(\hat{X_{0}},2\right)\right)$$

where $X_{0}$ is the wavelength data with random error added, $\hat{X_{0}}$ is the wavelength data without random error added, error is the assumed random error, and unifrnd(−1,1,1, size($\hat{X_{0}}$,2)) is a continuously uniform random number in the range of [−1,1]. The percentages of random error added are 0%, 1%, 3%, 5%, 10%, 15%, 20%, 25%, 30%, and 35%. The wavelength data with different degrees of random errors were imported into CNN, RF, KNN, and SVM for testing, and the accuracy of the three was compared, as shown in Figure 19.

It can be seen from Figure 19 that random error will have an impact on the classification recognition accuracy of the algorithm. As the proportion of random error increases, the accuracy of the algorithm decreases gradually. In the process of random error increasing from 0% to 5%, the accuracy of recognition results is maintained at a good level, and the relative errors of CNN, RF, KNN, and SVM are no more than 0.2%, 0.4%, 0.9%, and 1.3%. However, when the proportion of random error exceeds 5%, the error of recognition results increases significantly. The maximum accuracy relative error of CNN, RF, KNN, and SVM is 3.6%, 5.9%, 8.6%, and 10.17%. The results show that CNN has better recognition accuracy and noise resistance than the other algorithms.

In summary, if there are enough data to include various sizes of a certain shape, the contact surface shape of the contact object can also be better identified by CNN. Therefore, the shape recognition method based on FBG tactile sensing and CNN has a better ability to recognize different shapes, which also provides the basis for the detection of more complex shapes.

## 6. Conclusions

In this paper, the FBG tactile shape sensing array is made with 3D printing and flexible resin, and the classification recognition of 2D shapes is realized based on RF. The following conclusions are obtained:

1. The FBG sensing array made of 3D printing and flexible resin has the advantages of simple structure and wiring, convenient production, good anti-interference, etc. The shape recognition can overcome the defects of visual recognition by using the tactile form. When the adhesive length of FBG is 20 mm or above, the strain transfer rate of FBG can be well guaranteed; when the hole diameter of FBG is 0.2 mm, the strain transfer rate is higher.
2. The FBG tactile shape sensing array is sensitive to external load perception, and the overall change is relatively uniform. The average fitting advantage of FBG is above 99.8%, the average sensitivity of FBG with a grid length of 10 mm is 15.105 pm/N, and the average sensitivity of FBG with a grid length of 5 mm is 10.24 pm/N. The relative error of the sensitivity of loading and unloading a single FBG does not exceed 2%. The linearity of FBG is good, and its value is within 0.04.
3. CNN, RF, SVM, and KNN are used to classify and identify 2D shapes. CNN is better than the remaining three algorithms. Its accuracy is 6.11%, 9.44%, and 12.01% higher than RF, KNN, and SVM, and its F1 score is 6.3%, 8.73%, and 11.94% higher than RF, KNN, and SVM. The accuracy of CNN for the square, circle, rectangle, triangle, pentagon, hexagon, heptagon, octagon, etc. reaches 96.58%.
4. The number of shapes and the contact force will affect the recognition result. The higher the number of shapes, the lower the accuracy. For the same shape, the greater the contact force applied, the better the recognition result.
5. There is a negative correlation between the proportion of random error and algorithm accuracy. With the increase in the proportion of random error, the accuracy of the algorithm will gradually decrease. CNN has good recognition accuracy and noise resistance. After adding random error, the accuracy of CNN can still maintain a high level, and the relative error of its accuracy is less than 4%.

In the future, the classification and recognition algorithm, three-dimensional shape recognition, and sensing array analysis in the bending state are studied so as to better meet the requirements of flexible skin.

## Figures and Tables

**Table 1:** The parameters for the fabrication of the FBG sensing array.

Parameters	Maximum Strain Range/mm
Depth of embedding of FBG	[1.498, 3.502]
Optimal spacing of fiber	[14.989, 35.003]
Optimal spacing of grating	[31.25, 66.667]

**Table 2:** The parameters of the FBG.

Name of FBG	Wavelength/nm	Grating Length/mm	Wavelength Deviation/nm	Reflectivity	Side-Mode Suppression Ratio/dB
FBG1	1555.028	5	±0.5	>0.7	≥15
FBG2	1544.972	10	±0.5	>0.9	≥15
FBG3	1550.098	5	±0.5	>0.7	≥15
FBG4	1540.112	10	±0.5	>0.9	≥15
FBG5	1555.035	5	±0.5	>0.7	≥15
FBG6	1540.004	10	±0.5	>0.9	≥15

**Table 3:** Linearity of FBG.

Name of FBG	Linearity
FBG1	0.04
FBG2	0.008
FBG3	0.014
FBG4	0.008
FBG5	0.028
FBG6	0.009

**Table 4:** Information on the selected shapes.

Shape	Area/cm^2^	Mass/g	Material
triangle	15	3.2	resin
circle	16	3.9	resin
square	9	1.7	resin
rectangle	14	2.9	resin
pentagon	15.48	3.2	resin
hexagon	10.392	1.7	resin
heptagon	14.536	2.9	resin
octagon	13.91	2.7	resin

**Table 5:** Parameters of CNN.

Layer Connection	Input	Operation	Convolution Kernel	Output
0–1	3000 × 1	convolution 1	25 × 1 × 8	3000 × 8
1–2	3000 × 8	max pooling 1	15 × 1	200 × 8
2–3	200 × 8	convolution 2	25 × 1 × 16	200 × 16
3–4	200 × 16	max pooling 2	15 × 1	13 × 16
4–5	13 × 16	fully connection	208	208
5–6	208	fully connection	128	128
6–7	128	fully connection (softmax)	8	8

## Data Availability

The data presented in this study are available on request from the corresponding author.
